# Machine Learning in MRI Brain Imaging: A Review of Methods, Challenges, and Future Directions

**DOI:** 10.3390/diagnostics15212692

**Published:** 2025-10-24

**Authors:** Martyna Ottoni, Anna Kasperczuk, Luis M. N. Tavora

**Affiliations:** 1 Faculty of Mechanical Engineering, Bialystok University of Technology, 15-351 Bialystok, Poland; a.kasperczuk@pb.edu.pl; 2 Escola Superior de Tecnologia e Gestão (ESTG), Polytechnic of Leiria, 2411-901 Leiria, Portugal; luis.tavora@ipleiria.pt; 3 Instituto de Telecomunicações, Polytechnic of Leiria, 2411-901 Leiria, Portugal

**Keywords:** machine learning, magnetic resonance imaging (MRI), brain tumor, classification, brain imaging

## Abstract

In recent years, machine learning (ML) has been increasingly used in many fields, including medicine. Magnetic resonance imaging (MRI) is a non-invasive and effective diagnostic technique; however, manual image analysis is time-consuming and prone to human variability. In response, ML models have been developed to support MRI analysis, particularly in segmentation and classification tasks. This work presents an updated narrative review of ML applications in brain MRI, with a focus on tumor classification and segmentation. A literature search was conducted in PubMed and Scopus databases and Mendeley Catalog (MC)—a publicly accessible bibliographic catalog linked to Elsevier’s Scopus indexing system—covering the period from January 2020 to April 2025. The included studies focused on patients with primary or secondary brain neoplasms and applied machine learning techniques to MRI data for classification or segmentation purposes. Only original research articles written in English and reporting model validation were considered. Studies using animal models, non-imaging data, lacking proper validation, or without accessible full texts (e.g., abstract-only records or publications unavailable through institutional access) were excluded. In total, 108 studies met all inclusion criteria and were analyzed qualitatively. In general, models based on convolutional neural networks (CNNs) were found to dominate current research due to their ability to extract spatial features directly from imaging data. Reported classification accuracies ranged from 95% to 99%, while Dice coefficients for segmentation tasks varied between 0.83 and 0.94. Hybrid architectures (e.g., CNN-SVM, CNN-LSTM) achieved strong results in both classification and segmentation tasks, with accuracies above 95% and Dice scores around 0.90. Transformer-based models, such as the Swin Transformer, reached the highest performance, up to 99.9%. Despite high reported accuracy, challenges remain regarding overfitting, generalization to real-world clinical data, and lack of standardized evaluation protocols. Transfer learning and data augmentation were frequently applied to mitigate limited data availability, while radiomics-based models introduced new avenues for personalized diagnostics. ML has demonstrated substantial potential in enhancing brain MRI analysis and supporting clinical decision-making. Nevertheless, further progress requires rigorous clinical validation, methodological standardization, and comparative benchmarking to bridge the gap between research settings and practical deployment.

## 1. Introduction

The world has been experiencing a huge technological transformation in recent years, thanks to the development of ML [1]. Medicine, specifically the analysis of medical images obtained by MRI, is greatly influenced by machine learning [2]. Manual analysis of brain images is prone to errors and time-consuming [3]. Models capable of quickly analyzing large amounts of data are a very significant advantage when rapid diagnosis and treatment implementation in oncology patients are most valuable [2].

Despite significant progress in medical image analysis, automatic detection and segmentation of brain tumors remain a challenge [4]. This complexity arises from the limitations of manual methods, as manual segmentation is deemed unreliable in routine practice due to high inter-observer variability [5]. For automated models, difficulties stem primarily from the high variability of tumor appearance, particularly in terms of size, shape, intensity, and morphology [6]. This variability often results in ill-defined and irregular boundaries with surrounding healthy tissue [7]. Additional technical hurdles include the inherent problem of a deficient amount of labeled data for DL models [6], which tends to cause overfitting and poor generalization ability [6]. Furthermore, non-biological variations known as “scanner effects”, introduced by the use of different acquisition protocols and equipment, negatively affect model robustness and generalization capability [8]. Addressing these specific challenges is essential for developing reliable AI-driven diagnostic tools.

Khairandish et al. [9] present a hybrid CNN and support vector machine (SVM) model. The authors indicate that, despite high accuracy (98.5%), the challenge is the insufficient number of images for analysis.

Anaya-Isaza and Mera-Jiménez [10] present an approach based on data augmentation and transfer learning. The authors propose a comparison of different data augmentation methods and knowledge transfer strategies from other datasets, indicating the difficulties in obtaining adequate quality results from limited data. They note that one of the main challenges in medicine is the small number of samples, which makes it difficult for models to generalize and increases the risk of overfitting.

Alsubai et al. [11] present the application of ensemble methods in the diagnosis of brain tumors, using a combination of different algorithms, such as CNN–long short-term memory (CNN–LSTM), to improve classification performance. Ensemble methods, such as bagging or boosting, are used to combine the results of different models into one, which allows for more stable and accurate predictions. The authors note that such an approach is helpful, especially in the diagnosis of difficult cases, where a single model may not cope with the high variability of image data.

Sahaai et al. [12] presented the use of the binary robust invariant scalable keypoints (BRISK) descriptor for feature extraction from MRI images in the context of detecting brain tumors. This method is used in multi-class classification, where the authors use classifiers such as k-nearest neighbors (k-NN), SVM, and Random Forest (RF). However, they point out the challenge of the high number of false positives that can occur in the classification of tumors with similar texture and shape to healthy tissues.

Almalki et al. [13] present a method for feature extraction using isolated CNNs, which then use SVM classifiers to classify MRI images. Despite the high classification accuracy, the authors note the problem of the time required to train deep learning models, which is especially challenging for large datasets with images of varying quality.

Ali et al. [14] present a method for segmenting brain tumors using U-Net and 3D CNN in an ensemble approach. They use two networks to segment different tumor subregions, such as the growing tumor and the tumor core, and then combine the results. This approach achieved good results in the BraTS-19 challenge, with Dice coefficients of 0.750 for enlarging tumors, 0.906 for the entire tumor, and 0.846 for the tumor core. The authors highlight challenges related to tumor heterogeneity, blurred boundaries between the tumor and healthy tissue, and the variability of MRI images.

Several review papers have addressed the application of machine learning methods in brain MRI analysis [2,9]. Nevertheless, methodological advances and new architectures require an updated and focused review that reflects the current state of knowledge and unresolved challenges. The following studies offer relevant background and illustrate the need for an updated and focused review. A landmark review by Akkus et al. [15] analyzed the evolution of deep learning techniques in brain MRI segmentation, highlighting the transition from classical machine learning methods to fully convolutional neural networks. The study discussed various architecture styles, including patch-wise and semantic-wise CNNs, and emphasized their advantages in handling complex brain structures. Moreover, the authors outlined critical preprocessing steps, common evaluation metrics, and the limitations of existing datasets, thus providing a comprehensive foundation for subsequent algorithmic development in this domain.

In a more recent survey, Soomro et al. [4] provided an extensive review of MRI-based brain tumor segmentation approaches, spanning from traditional thresholding and region-based methods to advanced deep learning frameworks. Their analysis emphasized the growing dominance of convolutional and hybrid architectures, as well as the persistent challenges posed by data heterogeneity, class imbalance, and the high dependency on annotated ground truth. The authors also presented a structured evaluation of over 120 studies and highlighted the need for improved clinical generalizability and interpretability in segmentation pipelines.

Despite the significant advances in the use of machine learning techniques for the detection and segmentation of brain tumors in MRI images, several important gaps should be further addressed, such as: (i) many studies focus on specific machine learning algorithms, such as CNNs or SVMs, without directly comparing their performance on different datasets and different tumor types; (ii) insufficient attention is paid to data augmentation and its impact on model performance, although it is an important element in overcoming the problem of limited sample size; and (iii) generalization of models to real clinical data is still underexplored, as many studies are based on test sets that do not reflect the full diversity of patients [16]. These gaps indicate the need for further research to address these issues and improve the performance and usability of machine learning models in medical image analysis.

This literature review summarizes the current state of the art of ML-based approaches used in brain MRI analysis, including tumor detection and segmentation. It addresses key questions related to the most effective ML methods, limitations in data availability and model generalization, and potential directions for future research. The review discusses commonly used techniques, evaluating their effectiveness across different datasets. Furthermore, it highlights major challenges, including limited data, difficulties in generalizing models to clinical settings, and issues related to data augmentation. Based on this synthesis, areas requiring further research and development are identified to enhance the accuracy and clinical utility of these methods.

Compared to previous reviews on artificial intelligence and machine learning in neuroimaging, this study provides an updated and focused synthesis of the literature published between 2020 and 2025. It integrates both classical machine learning and state-of-the-art architectures, including hybrid and transformer-based models, reflecting the latest methodological progress. The review also presents a structured overview of key studies, summarizing their datasets, preprocessing techniques, and evaluation metrics to highlight current trends and research gaps. Furthermore, special emphasis is placed on emerging issues such as data imbalance, reproducibility, and the growing role of AutoML approaches in brain MRI analysis.

The remainder of this paper is organized as follows:

- Section 2 presents the literature review methodology, including the search strategy, databases used, and inclusion and exclusion criteria.
- Section 3 focuses on the datasets used to train and evaluate machine learning models, including data quality, labeling, and the availability of public datasets.
- Section 4 reviews common machine learning methods used in brain MRI analysis and discusses their performance. It also provides examples of their applications in tumor detection, segmentation, and classification.
- Section 5 identifies challenges and future research directions, in particular limitations related to data generalization and improving model robustness for clinical use.
- Section 6 presents the discussion, providing a critical interpretation of the reviewed literature and highlighting open issues.
- Section 7 concludes the paper with a summary of key observations.

## 2. Materials and Methods

This review focused on studies applying machine learning to brain tumor analysis using MRI. Searches were conducted in Scopus, PubMed, and the Mendeley Catalog (MC)—a public, read-only bibliographic catalog accessible via the Mendeley API and linked to Elsevier’s Scopus indexing system [17]. The search strategy used relevant keywords such as “brain tumor analysis”, “deep learning”, “machine learning”, “texture analysis” and “brain”, combined with Boolean operators (e.g., (“brain tumor analysis” AND “deep learning”) OR (“machine learning” AND “texture analysis” AND “brain”)). Only articles published in English between January 2020 and April 2025 with full-text availability, regardless of whether access was open or provided through instututional login.

Inclusion criteria were: studies involving human patients with brain tumors, use of MRI as the primary imaging method, a clear machine learning application, and documented validation (defined as a reported model evaluation procedure, including either internal validation such as k-fold cross-validation or train/test split, or external validation on an independent dataset). Excluded were studies without imaging data, animal studies, those lacking proper validation or statistical analysis, publications before 2020, non-English articles, and inaccessible full texts. In cases where full texts were not accessible through open access or institutional login, the authors attempted to obtain them when possible. When access was not granted, the studies were excluded and replaced by comparable accessible papers to minimize selection bias.

From 2178 records retrieved (MC 468, Scopus 1213, PubMed 497). After removing 583 duplicates, 1519 records remained for screening. Title and abstract screening led to the exclusion of 1202 records that did not meet the inclusion criteria. Subsequently, 393 full-text articles were evaluated for eligibility, and 108 met all criteria and were included in the final analysis.

To improve linguistic clarity, the manuscript was edited using ChatGPT (GPT-5, OpenAI) exclusively for grammar and style suggestions. No part of the scientific content, data analysis, or interpretation was generated by AI. All scientific content, analysis, and conclusions were developed and verified by the authors.

## 3. Datasets for MRI Brain Image Analysis

The effectiveness of machine learning models in brain MRI analysis heavily depends on the quality, diversity, and availability of datasets used for training and evaluation. Publicly accessible datasets play a crucial role in benchmarking methods, enabling reproducibility, and accelerating research progress. However, challenges such as class imbalance, limited sample sizes, inconsistent labeling, and data heterogeneity still pose significant limitations. This section provides an overview of the most widely used brain MRI datasets, their characteristics, and related issues in data acquisition and annotation.

### 3.1. Data Quality, Labelling Issues, and Dataset Limitations

One of the main challenges facing databases is the scarcity of labeled datasets. Acquiring and reliably labeling data is time-consuming and requires collaboration with clinical experts [8]. Limited datasets pose a risk of model overfitting [18].

Another problem is the imbalanced classes in the available datasets [19]. Less common tumor types are underrepresented, so the model, despite high accuracy, may fail to recognize them [19]. From a clinical perspective, the failure to recognize an aggressive, sporadic tumor is a serious limitation in medical diagnostics [19].

Furthermore, MRI data are characterized by significant heterogeneity and a lack of standardization across medical institutions [8]. Varying scanner settings, such as magnetic field strength and image resolution, as well as differences in acquisition protocols and MRI sequence types, lead to significant differences in signal quality and intensity [8]. These inconsistencies make it difficult to standardize the data, which means that models trained on data from one facility do not achieve comparable results on data from other facilities [6].

### 3.2. Comparative Overview of Datasets

Table 1 presents a summary of the datasets most frequently used in the reviewed studies. The table includes key characteristics such as imaging modality, dataset source, and the number of images, facilitating a comparative evaluation of dataset quality and suitability for brain tumor analysis using machine learning techniques.

The most commonly used dataset was BraTS (Brain Tumor Segmentation Challenge), used in seven of the reviewed studies (e.g., Yang et al. [34] Amin et al. [23], Ullah et al. [36]). BraTS contains multimodal images (T1, T1c, T2, FLAIR) with expert segmentations of tumor regions and is standardized in terms of preprocessing (registration, skull-stripping, normalization), making it a valuable resource for segmentation and classification tasks.

In addition to BraTS, many studies also relied on datasets shared through public repositories such as Kaggle and Figshare, which are popular sources particularly for classification tasks due to their broad accessibility. Unfortunately, these databases are usually limited to 2D slices and do not contain complete spatial orientation information or clinical metadata, which makes their application to 3D models difficult and limits their reliability in research. Some studies used clinical data from institutions such as Nanjing Brain Hospital [25], Hallym University [24] or Tianjin Medical University [32]. Although these data better reflect real clinical conditions, they are often characterized by greater heterogeneity (different scanners, imaging protocols) and a lack of standardized segmentations.

In summary, the choice of dataset, given its specific characteristics, may significantly constrain a general assessment of algorithms’ performance. Data from BraTS was found to be preferred in segmentation tasks, while datasets from Kaggle and Figshare in three-class classification (glioma, meningioma, pituitary). The problem remains the lack of class balance (overrepresentation of gliomas) and the limited number of real-world clinical cases available for analysis.

While the majority of reviewed studies relied on conventional MRI sequences (T1, T2, FLAIR, T1c), recent research increasingly emphasizes the importance of advanced functional modalities such as Diffusion-Weighted Imaging (DWI) and Perfusion-Weighted Imaging (PWI) [49,50]. DWI-derived parameters, such as the apparent diffusion coefficient (ADC), reflect tumor cellularity, whereas perfusion metrics (rCBV from DSC and Ktrans from DCE) provide insights into vascular proliferation [49]. Studies employing these multimodal inputs, including both retrospective prospective clinical cohorts, demonstrate that incorporating DWI and PWI features enhances prognostic modeling and supports more accurate tumor grading [49].

## 4. Machine Learning Techniques in MRI Brain Image Analysis

ML techniques used in MRI image analysis can be divided into supervised and unsupervised [51]. Supervised learning involves training data that includes ground truth annotations, such as class labels (for classification) or segmentation masks (for image delineation). These methods include SVM, RF, CNN [51].

Unsupervised learning, which includes clustering or autoencoders, is a method in which the data is not labeled [52]. In MRI image analysis, this method is less commonly used because it requires precise labeling of anatomical structures. However, it can be used to discover patterns of features or anomalies. Given their relevance, the review carried out focuses on supervised learning, which dominates current research [53]. It presents approaches described in the scientific literature on the detection, classification, and segmentation of brain tumors. The methods have been organized by type and complexity—from classic supervised learning algorithms, through deep neural networks, to ensemble approaches.

### 4.1. Classical Machine Learning Methods

#### 4.1.1. Support Vector Machines

Support Vector Machine (SVM) is a classical supervised learning algorithm based on statistical learning theory. It aims to find the optimal hyperplane that maximally separates data samples of different classes in a high-dimensional space. SVM are often used in medical imaging due to its generalization capabilities, especially in situations involving limited or high-dimensional data [54]. The algorithm’s flexibility is enhanced by the use of various kernel functions, such as linear, polynomial, radial basis function (RBF), and quadratic kernels, which allow it to adapt to different data distributions [54]. In the context of brain MRI analysis, SVM is frequently employed after handcrafted feature extraction and can effectively distinguish between normal and abnormal tissue patterns.

Mandle et al. [20] proposed a hybrid method that combines SVM clustering for segmentation and classification of brain tumors in MRI images. Their approach includes basic preprocessing, k-means clustering for segmentation, and discrete wavelet transform (DWT) with principal component analysis (PCA) for feature extraction and selection. The study was conducted on a dataset of 160 T2-weighted images from AANLIB, OASIS, and Harvard Medical School. A classification accuracy of 98.75% and a Dice score of 0.94 for segmentation were achieved, confirming SVM’s effectiveness in hybrid models. Although deep learning hybrids are widely used, classical combinations such as clustering with SVM have also shown high accuracy. Feature extraction and selection techniques such as DWT and PCA play a key role in improving the performance of SVM in high-dimensional MRI data.

Wahlang et al. [21] also evaluated the performance of SVM as a baseline model for brain MRI classification. The SVM classifier achieved an overall accuracy of 91%, which was slightly lower than the best-performing LeNet CNN model in the same study.

Sahaai et al. [12] applied SVM for multi-class classification of brain tumors using features extracted with the BRISK descriptor. The authors tested different kernels and found that an RBF kernel performed the best. The method achieved an accuracy of 97.59% and sensitivity of 93.24%, outperforming kNN in the same setup.

Yu et al. [22] used an SVM classifier to distinguish glioma and meningioma in MRI images based on 25 extracted texture features. After feature selection using the Gini index [55] from a random forest model, the best five features were used to train the SVM. The classifier achieved an AUC of 0.932, a sensitivity of 94.04%, and a specificity of 92.3%, demonstrating high discriminative performance in the binary classification of tumors (i.e., distinguishing between two tumor types).

Ni et al. [25] tested a linear SVM for predicting Ki-67 expression levels in glioma patients using MRI-based radiomic features. The model achieved one of the highest accuracies among all tested classifiers (0.884 ± 0.031) and an AUC of 0.904 ± 0.046, confirming its robustness in binary classification of radiogenomic targets. The model relied on a reduced set of features selected through LASSO and correlation filtering, extracted from multi-modal MR images segmented with nnU-Net.

The reviewed studies demonstrate the flexibility and effectiveness of SVM in brain MRI classification tasks. Despite the rise of deep learning methods, SVM remains a competitive approach, particularly when combined with robust feature extraction techniques. The presented results reflect a wide range of datasets and preprocessing strategies, confirming the general applicability of SVM in neuroimaging.

#### 4.1.2. Random Forest

RF is a classical ensemble learning method aggregating multiple decision trees (DT) to improve classification performance and reduce overfitting. Sahaai et al. [12] implemented RF with 50 trees for the multi-class classification of brain tumors, including glioma, meningioma, and pituitary tumors, using features extracted from BRISK and image characteristics. The model achieved 99.62% accuracy, 99.16% sensitivity, and 99.75% specificity, outperforming both SVM and kNN in the same experimental setup.

Yu et al. [22] also employed an RF model for the classification of brain tumors based on texture features extracted from MRI images. In addition to serving as a classifier, RF was used to select the most relevant features via the Gini index. The final model, trained on the top-ranked features, achieved an AUC of 0.856, with sensitivity of 82.8% and specificity of 88.3%. Although its performance was slightly lower than that of SVM, the results support the effectiveness of ensemble-based approaches in distinguishing between glioma and meningioma.

In a separate study focused on radio genomics, Ni et al. [25] employed a RF classifier to predict the level of Ki-67 expression in gliomas using radiomic features extracted from T1, T2, T1c, and FLAIR images. Although it performed slightly below logistic regression (LR) and SVM, the RF model still reached a solid AUC of 0.882 and accuracy of 0.830. The method was also used for feature ranking, which helped identify the most predictive image-derived features.

#### 4.1.3. Other Classical Methods (k-NN, Naïve Bayes, Decision Tree)

The k-NN algorithm is a simple yet effective classification technique that assigns class labels based on the majority vote of the nearest data points. In their study, Sahaai et al. [12] used k-NN with BRISK-based and intensity/shape-based features to classify brain MRI images into four categories. The performance varied depending on the chosen distance metric, with classification accuracy ranging from 93% to 94% and sensitivity from 82% to 91%, slightly below that of SVM and Random Forest.

Jo et al. [24] proposed a radiomic-based approach for evaluating changes in meningiomas after radiation therapy using brain MRI images. After semi-automated segmentation, 1691 radiomic features were extracted and refined through feature selection. An RF algorithm was used to build the predictive model, achieving accuracy of 73% and an AUC of 0.79 in the validation set. The study demonstrated that radiomic features, especially texture and shape extracted from CE-T1WI and CE-FLAIR images, could effectively differentiate between untreated and radiated tumors, suggesting that RF classifiers are suitable for predicting treatment effects in meningiomas.

Ni et al. [25] also evaluated several additional classifiers, including NB, DT, LR, and multiple boosting-based models. The NB model achieved an AUC of 0.880 and an accuracy of 0.827. At the same time, DT performed less effectively with an AUC of 0.709 and an accuracy of 0.803, highlighting the limitations of simpler models when dealing with complex, high-dimensional radiomic data. In contrast, LR yielded the best overall performance in the study, with an AUC of 0.912 and accuracy of 0.881. Among the boosting methods, LightGBM (LGBM) reached an AUC of 0.874, followed by eXtreme Gradient Boosting (XGBoost) (0.865) and gradient boosting trees (GBT) (0.863), all delivering competitive results but falling short of LR and SVM. These findings emphasize the importance of model selection tailored to the structure of radiomic datasets.

In their comparative analysis, Gajula and Rajesh [39] evaluated the performance of several classical machine learning classifiers, including SVM, RF, k-NN, NB, and multilayer perceptron (MLP), for brain tumor classification using MRI images. Although the exact numerical results for these traditional classifiers were not detailed in the study, they were used as benchmarks against the proposed five-layer CNN, which outperformed all others with an accuracy of 98.6%. The classical methods were applied after feature extraction and dimensionality reduction using PCA, reinforcing their utility in baseline comparisons for model selection.

### 4.2. Deep Learning Architectures

#### 4.2.1. Convolutional Neural Networks

CNNs are widely used in brain MRI analysis, given their ability to automatically learn spatial hierarchies of features directly from image data. CNNs utilize convolutional layers to extract localized features, which are subsequently processed by pooling and fully connected layers for classification or segmentation tasks [7,18]. In brain tumor studies, CNNs have been employed for both binary and multiclass classification as well as for pixel-level segmentation, often outperforming traditional approaches when sufficient annotated data is available [39]. Architectures are typically adapted to medical data by integrating domain-specific preprocessing (e.g., modality fusion or intensity normalization), and increasingly, CNNs are used in weakly supervised or hybrid pipelines to compensate for limited ground truth availability [29,45]. The success of CNNs in this domain is also attributed to their compatibility with multimodal MRI input and flexibility in integrating handcrafted or frequency-domain features, such as those derived from Discrete Wavelet Transform (DWT) or superpixel-based localization strategies [29].

In the study of Wahlang et al. [20], several CNN-based architectures were tested, including LeNet, CNN-DNN, ResNet50. Among these, the LeNet-based CNN achieved the highest generalization accuracy of 94%, while ResNet50 and AlexNet performed significantly lower (both at 59%), possibly due to overfitting on the limited dataset.

Papadomanolakis et al. [29] developed three variants of CNNs: a classic CNN model, a version extended with wavelet transform (CNN-DWT), and a model using transfer learning with a VGG16 architecture (CNN-TL). The CNN-DWT model utilizes a three-level discrete wavelet transform (HAAR) and uses frequency coefficients as input. The architecture included convolutional layers, pooling, dropout, and dense layers with a ReLU activation function. The classic CNN processed 240 × 240 px images as raw pixel intensities, while the CNN-TL was adapted to the input requirements of the VGG16 model. The CNN-DWT model achieved the highest sensitivity of 1.00, with an accuracy of 97% and a precision of 95%. The classic CNN model achieved a similar accuracy of 0.97 but a lower sensitivity of 0.94. The transfer learning-based model was characterized by lower efficiency (accuracy 0.87, specificity 0.84), which indicates a higher efficiency of approaches trained from scratch, especially when combined with wavelet analysis.

Singh and Saxena [30] proposed a two-stage framework for brain tumor analysis using a 2D CNN for classification, followed by a hybrid segmentation method. The CNN was trained on 884 preprocessed MRI images (tumor and non-tumor classes), resized to 224 × 224 pixels, and normalized using min–max scaling. The architecture consists of 11 layers, including convolutional blocks with LeakyReLU activation, batch normalization, and max-pooling, finalized with fully connected dense layers and a Softmax classifier. The CNN achieved a classification accuracy of 98.89%, with training and validation accuracies nearly identical (98.01% and 98.00%, respectively). Following classification, a hybrid segmentation pipeline combined graph-based segmentation (Felzenszwalb method) with thresholding to accurately localize tumor regions. The segmentation performance was evaluated using Bfscore (up to 1.0) and Jaccard similarity (up to 93.86%), confirming strong agreement with manual ground truth annotations.

Pathak et al. [31] proposed a framework combining a CNN for brain tumor classification and a marker-based watershed segmentation algorithm for tumor localization. Initially, T1-weighted MRI images are input to the CNN to classify whether a tumor is present. If classified as positive, the image undergoes segmentation using a watershed algorithm followed by morphological operations such as erosion to refine tumor boundaries. The CNN model consists of two convolutional layers (with ReLU activation and max-pooling), followed by fully connected dense layers and a sigmoid output neuron for binary classification (tumor vs. no tumor). The architecture employed small 3 × 3 kernels to reduce complexity while retaining feature extraction capability. The model was trained on 240 MRI images and validated on 87 additional images. It achieved a training accuracy of 98% and a validation accuracy of 100% after 15 epochs using the Adam optimizer with a learning rate of 0.001.

Badža and Barjaktarović [32] proposed a custom CNN architecture designed to classify three types of brain tumors: meningioma, glioma, and pituitary tumors. Their approach utilized a dataset of 3064 T1-weighted contrast-enhanced MRI images collected from 233 patients and implemented data augmentation techniques (rotation and vertical flipping), resulting in an extended dataset of 9192 images. The network was built with 22 layers, including convolutional, ReLU, dropout, max pooling, and fully connected layers, and trained using an Adam optimizer in MATLAB R2018a. Two evaluation strategies were adopted: record-wise and subject-wise 10-fold cross-validation. The best performance (96.56% accuracy) was achieved with record-wise validation on the augmented dataset, while subject-wise validation yielded an accuracy of 88.48%, confirming the generalization capability of the model for unseen subjects. The architecture proved lightweight, with only 4.3 million weights and an average classification speed under 15 ms per image.

Khan et al. [18] proposed a CNN-based model for binary classification of brain MRI images into cancerous and non-cancerous categories. The study used a small dataset of 253 MRI images, enhanced through data augmentation (flipping, rotation, brightness) and preprocessing (Canny edge detection and cropping to focus on brain regions). A custom CNN architecture with 8 convolutional layers and ReLU activation was developed, trained with a batch size of 32 using the Adam optimizer and binary cross-entropy loss. To benchmark the model, three pre-trained architectures—VGG-16, ResNet-50, and Inception-v3—were fine-tuned using transfer learning on the same dataset. The proposed CNN achieved 100% accuracy, outperforming all other models: VGG-16 (96%), ResNet-50 (89%), and Inception-v3 (75%). The evaluation was based on accuracy, precision, recall, F1-score, and AUC, with the proposed CNN achieving perfect scores across all metrics, demonstrating the efficiency of custom-designed CNNs in low-data scenarios.

Younis et al. [7] developed a custom CNN trained from scratch to classify brain tumors in T1-weighted MRI scans. The model was trained on a public dataset containing meningioma, glioma, and pituitary tumor classes. After applying bias correction, normalization, and image enhancement techniques, the CNN achieved an accuracy of 96%, demonstrating the viability of traditional convolutional architectures for tumor classification.

Gajula and Rajesh [39] proposed a five-layer CNN for classifying brain tumors in T1-weighted MRI scans. Their model included image preprocessing with adaptive noise filtering, segmentation using global thresholding, and extraction of texture and statistical features. The CNN architecture comprised convolution, pooling, and dense layers, optimized using RMSProp and binary cross-entropy. The network achieved an accuracy of 98.6%, outperforming architectures such as AlexNet, GoogleNet, and VGG-16, and demonstrating the effectiveness of lightweight CNNs in tumor detection.

Abd El Kader et al. [41] proposed a differential deep-CNN model to classify MR brain images into normal and abnormal cases. The model introduced additional differential feature maps using fixed filters applied to conventional CNN outputs, thereby enhancing feature representation without increasing network depth. This approach improved directional pattern detection within brain MRIs, addressing classification accuracy issues common in medical image analysis. The architecture included five convolutional layers with intermediate pooling, and was implemented using TensorFlow and Keras on the TUCMD dataset, which comprised 25,000 images. The model achieved an accuracy of 99.25%, sensitivity of 95.89%, specificity of 93.75%, precision of 97.22%, and F1-score of 95.23%.

Yoo et al. [45] proposed a weakly supervised segmentation pipeline for brain tumors in MRI using a combination of deep superpixel generation and clustering. The model is trained with binary image-level labels (tumor present/absent) rather than pixel-wise segmentations, reducing the annotation burden. The pipeline integrates a classifier trained with RISE (Randomized Input Sampling for Explanation) to produce localization seeds, which are then used as pseudo-labels to guide simultaneous training of a superpixel generator (based on a modified AINet architecture) and a superpixel clustering model (based on ResNet-18). Training was performed using multimodal 2D slices (T1, T1c, T2, FLAIR) from BraTS 2020 (369 volumes), with additional testing on BraTS 2023 (886 images). The model achieved Dice 0.745 and HD95 of 20.8 on BraTS 2023 test data, outperforming existing weakly supervised methods such as CAM-S (Dice 0.646) and SAM (Dice 0.641). The study demonstrates that leveraging undersegmented seeds improves segmentation quality and reduces propagation errors, offering a scalable approach in limited-annotation contexts.

Yang et al. [34] proposed the Multi-scale U-Net (MUNet) architecture for brain tumor segmentation, combining a U-Net structure with advanced modules inspired by transformer-based architectures to improve spatial representation. The model integrates the Selective Decoupling—State Space Model (SD-SSM) block, which leverages a state-space formulation and selective scanning to effectively capture both global and local contextual features—an approach conceptually related to the global attention mechanisms used in transformers. In addition, the Selective Decoupling Convolution (SD-Conv) structure is introduced to reduce feature redundancy without increasing the number of parameters. MUNet was trained and evaluated on three public datasets: BraTS2020, BraTS2018, and LGG-TCIA, incorporating multimodal MRI images (T1, T1c, T2, FLAIR). Segmentation accuracy was assessed using Dice similarity coefficients for three tumor subregions: Enhancing Tumor (ET), Whole Tumor (WT), and Tumor Core (TC). On the BraTS2020 dataset, the model achieved Dice scores of 0.835 (ET), 0.915 (WT), and 0.823 (TC), while on BraTS2018, performance achieved 0.835 (ET, TC), and 0.901 (WT). The model also demonstrated excellent generalization on the LGG dataset, confirming its robustness across tumor types and data sources.

Dixon et al. [38] developed a hybrid classification architecture that integrates deep CNN features, Vision Transformer (ViT) representations, and handcrafted radiomic features (e.g., Haralick, LBP). The model processes T1-weighted MRI images from three public datasets (7023 images) and a local clinical dataset (64 patients) spanning four classes: glioma, meningioma, pituitary tumor, and normal brain. Features extracted by each module were fused using a weighted ensemble strategy and classified using an MLP. The method achieved over 99% accuracy, sensitivity, and specificity on both public and local datasets. Ablation studies confirmed that ViT-derived features contributed the most to the overall performance, demonstrating the value of global attention mechanisms in brain tumor classification.

Pacal et al. [2] proposed a brain tumor classification model based on the Swin Transformer, enhanced with residual MLP blocks and hybrid attention mechanisms. The architecture was trained on a large public dataset and optimized for four-class classification (glioma, meningioma, pituitary, and healthy). The model achieved an overall classification accuracy of 99.92%, significantly outperforming traditional CNN architectures. The authors highlighted the advantage of Swin’s hierarchical attention mechanism in modeling fine-grained features and spatial context at different scales.

#### 4.2.2. U-Net and 3D CNN

U-Net is a fully convolutional architecture widely adopted for medical image segmentation due to its encoder–decoder structure with skip connections, enabling precise localization and effective fusion of spatial and contextual features [5]. Various modifications of the original U-Net have been proposed to improve its performance in brain tumor segmentation, particularly in handling heterogeneous tumor structures and small lesion regions [34]. Enhancements such as multiscale attention mechanisms and compound-scaled encoders like EfficientNetB4 have been introduced to improve feature representation across different spatial resolutions [5]. Furthermore, 3D variants of U-Net are employed to leverage volumetric information inherent in MRI, allowing for better modeling of tumor morphology across slices. These 3D CNN-based architectures capture inter-slice dependencies and spatial continuity, proving especially useful in high-resolution MRI datasets and applications requiring fine-grained tumor boundary delineation [5].

Archana and Komarasamy [26] employed a U-Net architecture to segment brain tumor regions from T1-weighted MRI images. The preprocessing included converting images from .MAT to .PNG, followed by cropping and resizing. U-Net processed coronal, axial, and sagittal views to extract the tumor region, generating binary segmentation masks. These segmented outputs were subsequently used as input for the classification phase based on an ensemble model.

Preetha et al. [5] proposed a novel segmentation framework integrating a Multi-Scale Attention U-Net with an EfficientNetB4 encoder to improve brain tumor segmentation in MRI images. The model introduces multiscale convolutions (1 × 1, 3 × 3, 5 × 5) and residual attention blocks to enhance feature extraction and tumor boundary delineation. Standard preprocessing steps included CLAHE, Gaussian blurring, and normalization. The method was evaluated on the Figshare dataset (3064 CE-MRI images of glioma, meningioma, and pituitary tumors), achieving outstanding performance: Dice score of 0.9339, IoU of 0.8795, recall of 0.9103, and precision of 0.9657. The EfficientNetB4 backbone provided a balanced trade-off between accuracy and computational efficiency, outperforming other EfficientNet variants and state-of-the-art segmentation models.

#### 4.2.3. Transfer Learning and Data Augmentation

Transfer learning has become an essential strategy in deep learning-based brain tumor analysis, allowing models pre-trained on large datasets (e.g., ImageNet) to be repurposed for medical imaging tasks with limited labeled data. This is particularly useful in MRI-based applications, where obtaining sufficient annotated brain scans is costly and time-consuming. In this approach, the knowledge learned by a model on a source domain is transferred and fine-tuned to perform a related task in a target domain, improving performance and reducing training time and computational cost [10,56]. Deep transfer learning techniques often include model-based strategies such as freezing early layers of a CNN while adapting the final layers, or applying progressive learning methods to prevent catastrophic forgetting [56,57]. Additionally, data augmentation plays a key role by synthetically increasing dataset variability through operations like rotation, flipping, or scaling, further enhancing generalization and model robustness [10]. These techniques are crucial in addressing the domain-specific challenges of brain MRI data.

Afzal et al. [6] proposed a robust transfer learning framework for brain tumor classification based on the ResNet18 model, enhanced with a novel hyperparameter tuning strategy named CART-ANOVA. The architecture was adapted for both four- and seven-class classification schemes, covering tumor types such as glioma, meningioma, pituitary tumor, no tumor, and glioma subtypes (astrocytoma, glioblastoma, oligodendroglioma). Their approach used two MRI datasets (from Kaggle and Radiopaedia), with the first used for training/testing (80/20 split), and the second for post-validation. Preprocessing included median filtering and resizing. A key innovation was the CART-ANOVA mechanism, which systematically explored and statistically evaluated combinations of learning rates and batch sizes to optimize training. The ResNet18 model achieved testing accuracies of 99.65% (four-class) and 98.05% (seven-class) on the primary dataset, and 98.78% and 96.77%, respectively, on the external dataset, confirming its high generalization capacity.

Ullah et al. [35] proposed a deep learning-based decision support system for the binary classification of brain tumor presence using MRI data. Their framework included four models: a CNN trained from scratch, and three transfer learning models—VGG-16, VGG-19, and LeNet-5. Due to the initially imbalanced dataset of 4600 images (2513 tumor, 2087 no tumor), data augmentation techniques such as flipping, rotation, and cropping were applied to achieve class balance. The models were trained with optimized hyperparameters and evaluated based on several metrics. VGG-16 and VGG-19 achieved the highest accuracy of 99.24%, outperforming CNN from scratch (99.02%) and LeNet-5 (98.80%). All models demonstrated high reliability with macro average values of 0.99 across accuracy, precision, sensitivity, specificity, and F1-score. The study confirmed that deep learning models, particularly with transfer learning and proper augmentation, offer robust solutions for BT detection and classification.

Ullah et al. [36] proposed a fine-grained classification framework for brain tumor detection using nine pre-trained transfer learning (TL) models: Inceptionresnetv2, Inceptionv3, Xception, ResNet18, ResNet50, ResNet101, Shufflenet, Densenet201, and Mobilenetv2. The models were fine-tuned and tested on a Kaggle dataset comprising MRI scans of glioma, meningioma, and pituitary tumors. Data augmentation techniques such as rotation and translation were applied to improve model generalization. Inceptionresnetv2 achieved the highest classification performance, with an accuracy of 98.91%, precision of 98.28%, recall of 99.75%, and F1-score of 99.00%, outperforming both standalone and hybrid CNN–SVM models.

In the same study, Younis et al. [7] further explored transfer learning by fine-tuning a VGG-16 model and incorporating it into an ensemble classification framework. The dataset, consisting of 253 T1-weighted MRI images, underwent extensive preprocessing, including N4ITK bias field correction, amplitude normalization, edge suppression, and augmentation methods such as rotation, brightness variation, and resizing. The VGG-16 model achieved 98.5% accuracy 94.4% recall and 92.6% of F1-score, while the ensemble model yielded the best results with 98.14% accuracy, 91.54% F1-score, and 91.4% recall. These results emphasize the benefits of combining pre-trained networks with augmentation and ensemble strategies to improve diagnostic accuracy in small, imbalanced medical datasets.

Shah et al. [40] proposed a transfer learning-based method using the pre-trained EfficientNet-B0 model fine-tuned with additional fully connected layers for binary classification of brain tumor MR images. Their approach incorporated three-step preprocessing (grayscale conversion, Gaussian blur, high-pass filtering), followed by normalization and data augmentation using the Albumentations library. Augmentation involved geometric transformations such as rotations and flips to improve generalization and mitigate overfitting. The dataset included 3762 MR images, equally split between tumor and non-tumor cases, sourced from Kaggle and derived from BraTS2015 and TCIA. The fine-tuned EfficientNet-B0 model outperformed other architectures such as VGG16, ResNet50, InceptionV3, and Xception, achieving a validation accuracy of 98.87%, AUC of 0.988, precision of 0.989, and F1-score of 0.988, with the smallest model weight (16.8MB) and parameter size among all compared methods.

Kuraparthi et al. [46] proposed a deep learning framework for brain tumor classification using transfer learning with three pre-trained DCNN architectures: VGG16, AlexNet, and ResNet50. Feature extraction was performed by fine-tuning the convolutional layers of these models, while the final classification was conducted using an SVM instead of the standard Softmax layer. The system was trained and evaluated using two public datasets: Kaggle (253 MR images) and BraTS 2015 (332 MR images), both labeled as binary classification problems (normal/abnormal or LGG/HGG). Data augmentation techniques such as rotation, flipping, and translation were employed to mitigate overfitting. The best performance was achieved using ResNet50 with SVM, reaching 98.28% accuracy on Kaggle and 97.87% on BraTS when data augmentation was applied. The results highlight that combining transfer learning with SVM can outperform Softmax-based classification, and that accurate tumor detection is achievable even with limited data and no prior image segmentation.

### 4.3. Ensemble Methods

Ensemble learning methods combine multiple models to improve predictive accuracy, robustness, and generalization. These approaches are instrumental in medical imaging, where individual classifiers may struggle with the complexity and variability of data such as MRI brain scans [11,23]. Ensemble strategies such as boosting and bagging enhance performance by aggregating the predictions of several base learners, reducing overfitting, and increasing stability [20]. While hybrid models like CNN–SVM and CNN–LSTM dominate many deep learning pipelines [2], classical ensemble techniques such as AdaBoost and Bagging have also shown competitive performance in recent studies [4].

#### 4.3.1. Hybrid CNN-SVM and Other Hybrid Models

Biswas and Islam [27] proposed a hybrid model that integrates a custom deep convolutional neural network (CNN) with an SVM classifier to enhance the classification of brain tumors in MRI images. The preprocessing pipeline involved image resizing, noise reduction via anisotropic diffusion filtering, and contrast enhancement using adaptive histogram equalization. Additionally, various augmentation techniques (scaling, shearing, translation) were applied to improve generalization.

The CNN consisted of five convolutional layers and two fully connected layers. Instead of relying on a softmax output, the deep features extracted from the final fully connected layer were passed to a multiclass SVM implemented using MATLAB’s fitcecoc function. The proposed CNN–SVM model achieved 96% accuracy on the Figshare brain tumor dataset, outperforming both softmax-based CNN (95.42%) and transfer learning baselines such as AlexNet (93.05%), GoogLeNet (89.39%), and VGG16 (85.24%).

Hashemzehi et al. [28] proposed a hybrid model combining CNN with neural autoregressive distribution estimation (NADE) to classify brain tumors from T1-weighted contrast-enhanced MRI images. In the proposed approach, CNN was first used to extract deep features, and then a NADE-based module modeled the probability distributions of the extracted features for improved classification. The model was trained and tested on 3064 images comprising glioma, meningioma, and pituitary tumor cases. This hybrid CNN–NADE model achieved high classification accuracy, demonstrating that combining deep feature extraction with probabilistic modeling can be an effective strategy in situations where medical image data is limited.

Gunasundari and Bhuvaneswari [33] proposed a hybrid classification model combining a differential convolutional neural network (DCNN) and an SVM for brain tumor detection and classification. The system employed extensive data augmentation (rotations, flipping, shifting, shear mapping) to expand the dataset to 25,000 MRI images. Feature extraction was performed using local binary patterns (LBP) and independent component analysis (ICA), followed by classification using an SVM with optimized kernel parameters (linear, sigmoid, and RBF). The proposed method achieved an overall accuracy of 98.96% and sensitivity of 0.973, outperforming standalone CNN and SVM models. The framework was evaluated on a dataset comprising T1-weighted contrast-enhanced MRI images collected from two hospitals in China and focused on three tumor types: glioma, meningioma, and pituitary tumor.

Muthaiyan and Malleswaran [44] proposed a classification framework based on Bendlet transform and ensemble learning (BEL) for MRI brain image analysis. The Bendlet transform, a second-order extension of the Shearlet transform, allows for enhanced detection of curved edges in brain images by incorporating bending operations. Feature extraction was performed on statistically selected sub-bands using the t-test, generating Bendlet Co-occurrence Features (BCFs) and histograms of positive and negative Bendlet coefficients (HPBC, HNBC). The classification task was conducted using an ensemble of three classifiers: k-Nearest Neighbors (k-NN), Naive Bayes, and Support Vector Machine (SVM), where each classifier’s output was weighted according to its accuracy. The method was evaluated on 200 images from the public REMBRANDT dataset and achieved 99.5% accuracy in the initial classification stage (normal vs. abnormal) and 99% in the final stage (low-risk vs. high-risk). The system bypasses the need for image segmentation and demonstrates strong potential for real-time CAD applications in brain tumor diagnosis.

#### 4.3.2. CNN-LSTM

In a study by Kumar et al. [42], a hybrid architecture combining a Gaussian-Weighted Deep Convolutional Neural Network and Long Short-Term Memory (GWDeepCNN-LSTM) was proposed for brain tumor classification. The method begins with a Gaussian-weighted non-local mean filter applied to brain MR images to remove noise and enhance contrast. Image segmentation is performed using Hartigan’s clustering algorithm, which partitions the image based on pixel similarity measured with Hamming distance. Feature extraction involves calculating texture, intensity, and color descriptors from each segmented region. These features are passed to an LSTM classifier, where similarity is evaluated using the Schutz index. The system was trained and evaluated on a dataset of 253 MRI scans. Reported performance included an accuracy of 95%, sensitivity of 98.21%, specificity of 72.54%, and a false positive rate of 5%. The average tumor detection time was 11.4 ms.

In the study by Dhaniya and Umamaheswari [43], a CNN-LSTM hybrid model was proposed for binary classification of brain MR images. The workflow consisted of Wiener filtering for denoising, followed by five data augmentation techniques: cropping, rotation, zooming, CLAHE, and random rotation with panoramic stitching (RRPS). Tumor segmentation was performed using the APSO algorithm, a variant of PSO enhanced with oppositional-based learning to improve convergence. Feature extraction was handled by a 20-layer CNN composed of convolution, pooling, dropout, and fully connected layers, while temporal dependencies were modeled using an LSTM layer. Evaluation on MRI data from the UCI Machine Learning Repository demonstrated strong performance with 92.03% accuracy, 92.36% sensitivity, 91.42% specificity, 92.93% precision, and an F-measure of 94.3.

Montaha et al. [3] proposed a hybrid model combining a TimeDistributed (TD) Convolutional Neural Network and Long Short-Term Memory (TD-CNN-LSTM) for binary classification of gliomas into high-grade (HGG) and low-grade (LGG). The model processes four 3D MRI sequences per patient (T1, T1c, T2, FLAIR), normalized with min-max scaling and resized to 128 × 128 × 32 voxels. A TD wrapper is applied to all CNN layers to allow separate spatial processing of each sequence, followed by an LSTM layer that models temporal dependencies across sequences. An ablation study was conducted to optimize architectural components and hyperparameters. The final model used the Nadam optimizer (learning rate = 0.0001), batch size of 16, and binary cross-entropy loss. When evaluated on the external BraTS 2020 dataset, it achieved 98.90% accuracy, 98.95% precision, 98.78% recall, 99.15% specificity, 98.83% F1-score, and an AUC of 99.04%. A comparative 3D CNN baseline trained on individual sequences yielded inferior results, supporting the benefit of using all sequences jointly via the TD-CNN-LSTM framework.

#### 4.3.3. Other Team Approaches

In addition to deep hybrid models combining CNN with other architectures, more classical ensemble approaches are also used. Methods such as AdaBoost and Bagging, which use intelligent fusion of simpler classifiers, can be used in hybrid approaches that aim to reduce computational complexity [37]. In the context of brain MRI analysis, they have shown competitive results compared to more complex models, especially when using extensive feature extraction.

In the classification stage of their pipeline, Archana and Komarasamy [26] applied a bagging-based k-nearest neighbor (BKNN) ensemble to distinguish between glioma, meningioma, and pituitary tumors using the segmented regions produced by U-Net. Their approach relied on aggregating predictions from multiple KNN classifiers trained on bootstrap samples, with final outputs determined by majority voting. The BKNN model achieved an accuracy of 97.7%, outperforming both the baseline KNN (95.4%) and a benchmark AdaBoost + SVM combination (96.3%). Evaluation was conducted using confusion matrices and per-class sensitivity and specificity, confirming the ensemble’s reliability in multi-class classification.

Zahoor et al. [37] proposed a two-phase deep hybrid learning framework for brain tumor analysis using MRI images. In the first phase, a deep-boosted feature space was created by combining features extracted from four top-performing transfer learning-based CNNs (InceptionV3, ResNet-18, GoogleNet, and DenseNet201). These were integrated into an ensemble classifier (DBFS-EC) using majority voting from SVM, MLP, and AdaBoostM1 classifiers, achieving excellent performance in binary classification between tumor and healthy cases (accuracy: 99.56%, precision: 0.9991, recall: 0.9899, F1-score: 0.9945). In the second phase, a hybrid features fusion-based classification approach (HFF-BTC) was developed for multi-class tumor classification (glioma, meningioma, pituitary). This model combined dynamic features from the custom CNN architecture BRAIN-RENet with static HOG descriptors and used SVM for final classification, reaching 99.2% accuracy and 0.9909 F1-score. These results highlight the effectiveness of combining ensemble learning with hybrid feature spaces in both tumor detection and subtype classification.

### 4.4. Comparative Overview of ML Methods

The utility of ML and DL methods in MRI analysis for brain tumor diagnosis must be critically assessed based on established performance metrics and optimal application scenarios. The selection of the most effective algorithm—whether a classical method like SVM or RF, or a deep learning approach like a CNN—is dictated by key factors, including the size of the dataset, the specific classification task (e.g., multi-class vs. grading), and the available computational resources.

The leading performance is exemplified by optimization frameworks like ResNet18 coupled with CART-ANOVA hyperparameter tuning [6]. This model achieved an exceptional testing accuracy of 99.65% for four-class and 98.05% for seven-class classification on the initial test set. Crucially, this framework established strong generalization capabilities by maintaining high accuracy rates (98.77% and 96.77%) when validated on an entirely independent, external dataset (Source 2). In broader multi-class tumor classification (glioma, meningioma, pituitary), the pre-trained Inceptionresnetv2 model consistently outperformed other TL models, achieving the highest accuracy of 98.91% [36]. While CNNs offer immense precision, models require substantial resources. However, custom-designed CNN architectures are feasible options when computational efficiency is prioritized, boasting a compact size of 4.3 million weights and very fast execution speeds (averaging less than 15 ms per image) [32].

Hybrid models are ideal for combining the power of automatic feature extraction from DL with the reliable classification performance of robust ML algorithms, often succeeding where stand-alone DL models struggle due to limited data. In 3D classification tasks, the TD-CNN-LSTM model is optimally suited for classifying gliomas into HGG and LGG. This architecture achieves the highest test accuracy of 98.90% by processing all four MRI sequences (T1, T1c, T2, FLAIR) simultaneously as a single input [3]. For 2D multi-class tasks, utilizing CNNs exclusively for feature extraction, followed by SVM classification, has proven superior to using the Softmax layer alone. For example, a hybrid Deep CNN-SVM model achieved 96.0% accuracy, outperforming three Transfer Learning models (AlexNet, GoogLeNet, VGG16) in the same comparative study [27].

Classical ML classifiers (RF, SVM) excel in tasks that require highly granular analysis based on engineered features (radiomics), such as predicting local malignancy or specific marker expression. RF is demonstrably the best algorithm for predicting local glioma malignancy grade (WHO grade), achieving 96% accuracy (Cohen’s κ = 0.93) using advanced imaging inputs (T2, ADC, CBV, Ktrans) [48]. RF also showed superior performance in multi-class classification based on engineered features, yielding 99.62% accuracy when utilizing BRISK descriptors, outperforming both SVM (97.59%) and k-NN (93.65%) in that specific setting [12].

Linear SVM is the top-performing model for predicting the Ki-67 proliferation index in gliomas using radiomic features, achieving the highest accuracy of 0.884 (AUC 0.904) among several classical models tested [25]. In binary classification problems, the effectiveness of SVM and CNN models is greatly enhanced when input data is transformed using methods like the DWT. A comparison showed that CNN-DWT achieved 0.97 accuracy and 1.0 sensitivity in distinguishing glioma tumors from other pathologies, and the SVM-DWT model was significantly superior in performance compared to standard SVM using pixel intensities [29].

For the complex task of classifying LGG/HGG based on features extracted from the peritumoral edema region in fused MRI images, a tuned SGD classifier achieved the highest performance (Accuracy 0.96, AUC 1.0) using T1Gd + FLAIR fused images [58].

Table 2 presents a comparative overview of the most relevant machine learning methods applied to brain MRI analysis, including classification, segmentation, and hybrid approaches. The table summarizes the studies in terms of methodology, preprocessing techniques, evaluation metrics, and task type. This structured comparison enables a better understanding of the strengths and limitations of each approach and facilitates identification of the most effective solutions.

As summarized in Table 2, machine learning techniques employed in brain MRI analysis exhibit varying levels of effectiveness, depending on the specific task (classification or segmentation) and the quality of the input data. Classical methods, such as SVM and RF, prove valuable, particularly in situations involving limited or high-dimensional data, and serve as strong baselines for comparison. However, deep learning models, notably CNNs and their hybrid architectures incorporating attention mechanisms or LSTM units achieve superior accuracy. Their performance is significantly enhanced by the application of transfer learning and data augmentation strategies, which are crucial for mitigating limited data availability and improving generalization. Despite these substantial advancements, current solutions still contend with significant limitations, including challenges in generalization to real-world clinical data, issues arising from class imbalance in datasets, and a pervasive lack of standardization in imaging protocols. Consequently, the subsequent chapter will comprehensively address these main challenges and explore future directions for machine learning methods within practical clinical applications.

Although many reviewed studies report very high accuracies (often exceeding 99%), such results should be interpreted with caution. In particular, Khan et al. [18] reported 100% accuracy for a CNN model trained on only 253 augmented MRI images, illustrating how extremely small datasets can inflate performance estimates. These outcomes are frequently associated with limited dataset sizes, image-wise rather than patient-wise validation, or extensive data augmentation, all of which increase the risk of overfitting and reduce clinical generalizability. For example, Badža and Barjaktarović [32] reported a 96.56% accuracy under record-wise validation, which dropped to 88.48% when evaluated with subject-wise validation, demonstrating the impact of more rigorous testing protocols. Conversely, Afzal et al. [6] achieved 99.65% accuracy on the initial test set and maintained 98.78% performance on an independent external dataset, supporting better generalization. Therefore, validation strategy and dataset scale are crucial for assessing the true robustness of ML models in MRI-based brain tumor analysis.

In the context of the evolution of machine learning methods, an emerging trend is the application of AutoML, which aims to automate the process of building optimal ML pipelines, thereby increasing efficiency and reducing manual bias. Khorasani et al. [58] recently demonstrated the effectiveness of this approach for glioma classification by integrating radiomic features derived from peritumoral edema regions into an AutoML framework. The study employed the TPOT (Tree-based Pipeline Optimization Tool) for automated pipeline design and the Boruta algorithm for feature selection. The optimization process identified a fine-tuned Stochastic Gradient Descent (SGD) classifier trained on fused MRI sequences (T1Gd + FLAIR), which yielded superior performance compared to single-sequence models. This AutoML-based pipeline achieved 96% accuracy and an Area Under the Curve (AUC) of 1.0 for distinguishing LGG from HGG. These findings highlight AutoML’s potential to accelerate the development of robust and clinically reliable ML models by automating hyperparameter tuning and model selection.

## 5. Challenges and Future Directions

Contemporary advances in AI-assisted diagnostics open up new possibilities, but also present researchers with a number of significant challenges. The development of automated diagnostic systems requires not only achieving high accuracy but also ensuring their practical utility, interpretability, and resilience to clinical data variability.

### 5.1. Technical and Clinical Challenges

Although deep neural networks such as VGG16 and GoogLeNet offer high accuracy, their computational requirements and training time can be significant, often demanding dedicated hardware and hundreds or even over a thousand minutes per classification [32]. Simpler networks can run on conventional personal computers, consuming significantly fewer resources [32].

Despite impressive results, improving interpretability (XAI) remains a key step towards widespread adoption of AI systems in medical diagnostics [59]. Although inherently interpretable models are desirable, there is often an explainability-performance trade-off [60]. Simpler, more transparent models can underperform compared with complex black-box architectures, while post hoc explanations for black boxes introduce their own limitations [49]. Implementing techniques that increase model transparency is essential to building trust among radiologists and physicians, as it allows them to understand how the algorithm makes decisions [55]. Integrating advanced AI methods into existing clinical workflows also requires appropriate training for clinicians and adaptation of the systems themselves [5].

A significant limitation in the development and implementation of AI systems is the lack of high-quality, labeled medical data [52]. MRI images often contain discrepancies, such as magnetic field inhomogeneities or motion artifacts, which can lead to false intensity values and require appropriate correction methods [50]. Furthermore, unbalanced datasets, such as Figshare, can negatively impact classification performance [6,52].

The issue of data imbalance is the primary methodological hurdle in AI-based neuroimaging, often resulting from the rarity of certain conditions, such as specific tumor subtypes [2,37]. When data is highly imbalanced, DL models tend to exhibit a bias toward the majority class, resulting in poor performance for the minority group [38]. To mitigate this problem, several data-level strategies are employed. Data augmentation (e.g., rotation, flipping) is commonly used to artificially expand small datasets, improving generalization and reducing overfitting [5,32]. More advanced techniques include oversampling methods such as SMOTE, which synthetically generate minority class instances [7], and the use of Generative Adversarial Networks (GANs) to produce realistic MRI samples for underrepresented classes [19].

Algorithmic approaches, including Cost-Sensitive Learning (CSL) and class weighting, assign greater importance to minority class errors [7]. Class weighting is one technique utilized to improve model performance in the presence of imbalanced data [7]. Specialized loss functions offer further refinement: Focal Loss (FL) is highly effective for extreme class imbalance in classification [7]. Furthermore, in segmentation tasks, where imbalance exists between foreground (tumor) and background pixels, specialized loss functions like the Dice Loss are frequently utilized [61]. Dice Loss is crucial for maximizing the overlap between prediction and ground truth, ensuring robust performance [61]. Combining these data-level and algorithm-level methods (hybrid approaches) is essential for developing robust diagnostic models capable of balanced performance across tumor categories.

The limited generalization ability of models trained on data from a single source also poses a challenge. These models may struggle to transfer their performance to previously unseen datasets or to images from other modalities [6,32]. Maintaining high accuracy across medical facilities with different MRI equipment and imaging protocols is a significant practical challenge [6,29].

An additional challenge in brain tumor classification is the difficulty in distinguishing between certain types. For example, meningiomas can be difficult to distinguish from other lesions due to their location and nonspecific imaging features [32].

It is also worth emphasizing that the current standard in histopathological diagnosis remains biopsy, which, although reliable, is an invasive procedure and carries a risk of complications such as bleeding, tissue damage, or even loss of neurological function [3]. In this context, AI systems may provide a less invasive diagnostic alternative [3,38].

### 5.2. Emerging Trends and Potential Improvements

Transfer learning and self-supervised learning methods are particularly valuable when dealing with limited medical datasets [62]. Pre-training on large natural image datasets (e.g., ImageNet) or unlabeled medical data can significantly improve the performance of medical models [62]. Transfer learning helps to avoid overfitting and reduces the need for large, manually labeled datasets [6]. Continued research should focus on utilizing diverse and larger datasets, including multi-center data and multi-modal imaging such as T1, T2, and FLAIR sequences [3,6]. Developing methods that not only classify but also quantify tumor characteristics, such as size and shape, is crucial for treatment planning [6]. Computer-aided diagnostic (CAD) systems also support prognosis, diagnosis, and pre- and postoperative decision-making [6,38]. Future work should aim to reduce the computational complexity of advanced models and further optimize them for real-time clinical applications [3,6,32]. Techniques like model pruning and dimensional extension can help decrease the consumption of graphical processing units [32]. Finally, efforts should focus on seamless integration of AI systems into existing clinical workflows, which requires clinician training and system adaptation to new tools [6].

### 5.3. Open Questions and Research Gaps

Practical interpretability of models remains an open question despite progress, as it is still unclear how to ensure that the interpretations provided by AI models are truly useful and understandable for clinicians in practice [28]. There is a crucial need for further validation of models across different MRI sequences and other independent datasets to guarantee their robustness and applicability in diverse clinical settings, regardless of scanner settings or location [3,6,14]. Research should also assess the impact of various preprocessing and image harmonization methods on radiomic features [14]. Ethical aspects and data security require continued investigation, including patient privacy, data protection, and potential model bias [32]. Integrating AI models with tumor growth models could provide more comprehensive insights into disease progression. Lastly, advancing AI toward more personalized treatment plans based on detailed tumor characterization remains a promising area for future research [61].

## 6. Discussion

This literature review aimed to provide a comprehensive overview of ML applications in the analysis of MRI of the brain, with a particular focus on tumor classification and segmentation. The analysis confirms that machine learning, especially deep learning methods, demonstrates significant potential in improving brain image analysis and supporting clinical decision-making.

The results of the review clearly indicate the dominance of CNNs and hybrid architectures in MRI image analysis. CNNs are widely used due to their ability to automatically extract relevant spatial features without the need for manual extraction. Moreover, hybrid architectures such as CNN-SVM [9,27] or CNN-LSTM [11,42,43] regularly achieve better results compared to single models. Also noteworthy are Transformer models such as Swin Transformer, which achieve very high classification accuracy [2], as well as non-standard CNN architectures, which have demonstrated 100% accuracy in binary classification tasks [18].

Despite the impressively high accuracy rates (often exceeding 99%) reported in many studies, a critical assessment of their practical utility and generalizability is necessary. One of the main methodological limitations remains the small size of the datasets, which, combined with excessive augmentation, leads to overfitting and can artificially inflate performance estimates. Furthermore, the use of image-level validation instead of patient-level validation masks actual generalization issues—in one case, accuracy dropped from 96.56% (image-level validation) to 88.48% (patient-level validation) [32]. It is also necessary to address the potential risk of data leakage that can occur when training and test data are not properly separated, especially in studies based on data from single centers. Work that has maintained high accuracy on independent, external datasets, such as the 98.78% accuracy in the study by Afzal et al. [6], is the gold standard for assessing model generalization ability.

Due to the limited availability of high-quality, labeled medical datasets, data augmentation and transfer learning play a key role. Both techniques improve model generalization and reduce the risk of overfitting [10]. Pre-trained models on large natural datasets (e.g., ImageNet) or unlabeled medical data has proven particularly effective.

The effectiveness of ML models is closely linked to the quality and diversity of the datasets. The most commonly used dataset is BraTS, which provides multimodal images with expert segmentation [34]. Data from platforms such as Kaggle and Figshare are also used, but they often lack spatial orientation information or clinical metadata, limiting their usefulness in generalizing results [36]. A significant problem remains the effectiveness of model generalization to data from different institutions, which stems from the heterogeneity of MRI data and the lack of standardization of imaging procedures [6]. As a result, models trained on a single data source may not achieve comparable results on data from other institutions [14].

Technical and clinical challenges also remain. Deep learning models require significant computational resources [13]. Limited interpretability is a barrier to their reliable use in clinical practice [28]. Additional challenges arise from limitations in available data, such as a lack of labels, class imbalance, and difficulty distinguishing certain tumor types.

To enhance transparency, various explainability techniques are employed. Popular methods include Gradient-weighted Class Activation Mapping (Grad-CAM) and Local Interpretable Model-agnostic Explanations (LIME) [59]. Grad-CAM visually highlights image regions that have the greatest impact on model predictions, which can be achieved by integrating with the ResNet50 architecture. Although warmer colors in heatmaps indicate greater importance of a given region, these techniques are not yet fully reliable for clinical diagnostic decision-making. It has been noted that Grad-CAM visualizations cannot accurately represent certain MRI images, suggesting the need for further refinement of the model architecture, hyperparameter tuning, or exploration of more advanced attention mechanisms. Researchers also face a general “explainability-performance trade-off,” where complex “black-box” models are more accurate but less transparent, and additional explanations (post hoc explanations) introduce their own limitations.

Ethical and bias issues are critical in the pursuit of integrating AI systems into clinical diagnostics. These issues, including fairness and transparency, are directly related to the limitations of available data [5]. Class imbalance is a dominant methodological obstacle, stemming from the rarity of certain tumor subtypes [35]. When data are highly imbalanced, DL models often exhibit bias toward the majority class, leading to poor performance in the minority group. Clinically, failure to diagnose aggressive, sporadic tumors is a serious limitation [35]. To mitigate this problem, data-level strategies such as data augmentation or more advanced methods such as generative adversarial networks (GANs) are used to generate realistic MRI samples for underrepresented classes [29,35]. Algorithmic strategies include class weighting or specialized loss functions such as Dice Loss, which are crucial in segmentation tasks due to the imbalance between background and tumor pixels [25,57]. Additional challenges arise from the lack of standardization in imaging protocols, introducing nonbiological differences known as “scanner effects,” which negatively impact the robustness and generalizability of the model, compromising the fairness of results across institutions [50].

In summary, despite significant progress in the use of ML for brain MRI analysis, further research must focus on improving model generalization, interpretability, computational efficiency, and standardization of analytical protocols. At the same time, ethical considerations and the integration of AI solutions into clinical diagnostic practice are essential.

## 7. Conclusions

Machine learning, particularly deep neural networks, has demonstrated high performance in the classification and segmentation of brain tumors in MRI images. Dominant architectures such as CNNs, hybrid models, and transformers achieve very high diagnostic accuracy, confirming their potential for clinical applications.

For classification tasks, the most effective architectures were those employing attention mechanisms and hybrid learning strategies. The Swin Transformer proposed by Pacal et al. [2] achieved the highest reported performance, reaching 99.92% accuracy across glioma, meningioma, pituitary, and healthy classes. This model utilized residual MLP blocks and hybrid attention mechanisms and was trained on a large public Kaggle dataset composed of three merged sources—Figshare, SARTAJ, and Br35H—including 7023 MRI images. Similarly, a custom CNN developed by Khan et al. [18] achieved 100% accuracy in binary classification (cancerous vs. non-cancerous) using the Kaggle (Navoneel) dataset comprising 253 MRI images. However, the small dataset size suggests possible overfitting and limited generalization.

Among hybrid approaches, the DBFS-EC proposed by Zahoor et al. [37] achieved 99.56% accuracy by combining features from four transfer learning CNNs (InceptionV3, ResNet-18, GoogleNet, DenseNet201) and training on a combined Kaggle–Figshare dataset (5058 images). Another highly efficient framework, developed by Afzal et al. [6], combined ResNet18 with CART-ANOVA-based hyperparameter optimization. This model achieved 99.65% accuracy in four-class and 98.05% in seven-class classification when trained on Kaggle and externally validated on Radiopaedia, demonstrating strong generalization capability across independent datasets.

For segmentation tasks, the best-performing models were U-Net-based architectures enhanced with attention mechanisms or advanced encoders. The Multi-Scale Attention U-Net with an EfficientNetB4 encoder (Preetha et al. [5]) achieved a Dice coefficient of 0.9339 and an IoU of 0.8795 on the Figshare dataset (3064 CE-MRI images), performing three-class segmentation of tumor subregions.

The MUNet model (Yang et al. [34]), integrating U-Net with state-space model blocks, achieved a Dice score of 0.915 for the Whole Tumor (WT) region on BraTS2020, confirming strong segmentation accuracy. Likewise, Ali et al. [14] reported robust performance using an ensemble of U-Net and 3D CNN architectures, obtaining a Dice coefficient of 0.906 in the BraTS-19 challenge. Key challenges remain, however. These include limited data availability, difficulties in generalizing results, high computational requirements, and limited model interpretability. Future research should focus on clinical validation, increasing the diversity of training data, reducing model complexity, and developing standards for evaluation and process standardization.

Ultimately, despite significant technological advances, the path to widespread implementation of ML-based solutions in everyday diagnostics requires further work on their practical usability and integration with real-world clinical environments.

Furthermore, improving the reproducibility and transparency of future studies remains a critical priority. To enhance methodological consistency, researchers are encouraged to adopt established reporting standards such as the CLAIM (Checklist for Artificial Intelligence in Medical Imaging) and CONSORT-AI guidelines. Following these frameworks, together with openly sharing code, dataset partitions, and preprocessing protocols, would substantially improve the comparability, reproducibility, and clinical translation of machine learning models in neuroimaging.

## Figures and Tables

**Table 1:** Summary of datasets used in brain MRI studies.

No.	Study	Acquisition Method	Dataset Sources	Data Split/Validation Method
1.	Mandle et al. [20]	T2-weighted MRI, axial plane, 256 × 256 px	AANLIB, OASIS, Harvard Medical School—160 images (20 normal, 140 abnormal)	5-fold cross-validation (129 Train, 32 Validation/Test)
2.	Wahlang et al. [21]	Multimodal MRI (FLAIR, T1-weighted), 2D axial slices	Figshare, Brainweb, Radiopaedia—2530 images (806 normal, 1534 abnormal)	5-fold and 8-fold cross-validation; generalization test on 506 images
3.	Sahaai et al. [12]	MRI, modality not reported, 2D slices	Kaggle—3264 images (500 normal, 926 glioma, 937 meningioma, 901 pituitary)	2870 Training/394 Testing
4.	Yu et al. [22]	Coronal MRI (modality not reported)	Clinical dataset, Nanjing Brain Hospital—80 regions (40 tumors, 40 healthy regions)	70% Training/30% Testing (iterated 5 times)
5.	Amin et al. [23]	Multimodal MRI (T1, T2,T1c, FLAIR)	BRATS (2012–2015), ISLES (2015)—535 cases (80, 30, 191, 274 cases of HG/LG Glioma)	5-fold cross-validation and 0.5 hold-out validation
6.	Jo et al. [24]	Multimodal MRI (T1WI, T2WI, CE-T1WI, FLAIR, CE-FLAIR), slice thickness 1 mm	Clinical dataset, Hallym University Sacred Heart Hospital (South Korea)—162 patients (meningiomas)	Random stratified sampling: 118 Training/44 Validation; 10-fold cross-validation (for radiomics features)
7.	Ni et al. [25]	Multimodal MRI (T1, T2, T1CE, FLAIR)	Clinical dataset, First Affiliated Hospital of Nanjing Medical University—613 patients (glioma)	10-fold cross-validation
8.	Archana & Komarasamy [26]	T1-weighted MRI, coronal/axial/sagittal planes	Figshare (Cheng)—3064 images (233 patients; 1426 glioma, 708 meningioma, 930 pituitary)	80% Training/20% Testing
9.	Biswas & Islam [27]	T1-weighted MRI, axial/coronal/sagittal planes, 512 × 512 px	Figshare—2957 images (1330 glioma, 697 meningioma, 930 pituitary)	80% Training/20% Testing
10.	Hashemzehi et al. [28]	T1CE MRI, 512 × 512 px	Clinical dataset—3064 images from 233 patients (708 meningioma, 1426 glioma, 930 pituitary); tumor boundaries manually annotated by a radiologist	6-fold cross-validation
11.	Papadomanolakis et al. [29]	T2-SWI MRI, 1.5 T scanner; 240 × 240 px (JPEG)/512 × 512 px (NIfTI), axial plane	St. George Hospital, BraTS (2016–2017), ISLES—572 T2 MRIs	5-fold cross-validation (382 Training/190 Testing)
12.	Singh & Saxena [30]	Multimodal MRI (T1, T2, FLAIR, T1c), axial/coronal/sagittal planes	Clinical dataset, Safdarjung, Medanta, SGPGI Hospitals (India)—884 images (624 tumor, 260 no-tumor)	80% Training/20% Testing (707/77 images; total dataset size inconsistently reported as 884)
13.	Pathak et al. [31]	T1-weighted MRI, axial slices	Clinical dataset, 5 medical centers w Surat (India)—327 images	240 Training/87 Validation
14.	Badža & Barjaktarović [32]	T1c MRI, sagittal/axial/coronal planes	Nanfang Hospital, Tianjin Medical University—3064 images (233 patients) (708 meningioma, 1426 glioma, 930 pituitary tumor)	10-fold cross-validation (60% Train, 20% Validation, 20% Test)
15.	Khan et al. [18]	MRI, modality not reported	Kaggle (Navoneel, 2019)—253 images (155 malignant, 98 benign)	185 Training/48 Validation/20 Testing
16.	Gunasundari & Bhuvaneswari [33]	T1C MRI (2D slices), 512 × 1024, slice thickness 6 mm	TUCMD dataset; collected from General Hospital of Tianjin Medical University and Nanfang Hospital (China); 2084 original images expanded to 25,000 after augmentation (7000 normal, 9000 abnormal; 3 tumor types: glioma, meningioma, pituitary)	5-fold cross-validation (~1800 abnormal/1400 normal per fold)
17.	Yang et al. [34]	Multimodal MRI (T1, T1c, T2, FLAIR) 240 × 240 × 155 px	BraTS2020 (369 images), BraTS2018 (285), LGG-TCIA (3929 MRI slices from 110 LGG patients)	5-fold cross-validation; independent test on BraTS2020 subset
18.	Afzal et al. [6]	T1 MRI, axial plane	Source 1: Kaggle, Radiopaedia—3137 images (4 and 7 classes). Source 2: Radiopaedia, Kaggle—1365 images (validation)	Source 1: 80% Training/20% Testing; Source 2: External Validation (Generalization Test)
19.	Ullah et al. [35]	MRI, modality not specified	Kaggle—4600 images (2513 tumor, 2087 no tumor)	70% Training/20% Testing/10% Validation
20.	Ullah et al. [36]	T1-weighted MRI	Kaggle—2475 images (822 meningioma, 826 glioma, 827 pituitary)	80% Training/20% Testing
21.	Younis et al. [7]	T1-weighted MRI	Brain MRI Images for Brain Tumor Detection—253 images (155 patients; meningioma, glioma, pituitary)	80% Training/10% Validation/10% Testing
22.	Zahoor et al. [37]	T1c MRI	Kaggle (normal) + Figshare (tumor types: glioma, meningioma, pituitary); total 5058 images (1994 normal, 3064 tumor)	Detection Phase: 60% Training/40% Testing; Classification Phase: 80% Training/20% Testing
23.	Preetha et al. [5]	T1c MRI; axial/sagittal/coronal planes; 512 × 512 resolution	Figshare dataset (3064 slices from 233 patients; glioma, meningioma, pituitary tumor)	90% Training/10% Testing
24.	Dixon et al. [38]	Multimodal MRI (T1, T2, FLAIR) 2D axial slices	Public datasets: Figshare, SARTAJ, Br35H—7023 images (1645 meningioma, 1621 glioma, 1757 pituitary, 2000 normal); Local dataset: Mansoura University Hospital—64 patients (normal, benign, malignant)	5-fold cross-validation; 80% Training/20% Testing; external validation on local dataset
25.	Gajula & Rajesh [39]	T1-weighted MRI	Custom dataset—3264 MRI images divided into 4 classes: glioma, meningioma, pituitary, and no tumor	2870 Training/394 Testing
26.	Shah et al. [40]	T1-weighted MRI	Kaggle (BraTS 2015, TCIA)—3762 images; subset of 3060 used (1500 labeled as tumor, 1500 as no tumor)	80% Training/20% Validation
27.	Abd El Kader et al. [41]	Multimodal MRI (T1, T2, FLAIR)	TUCMD dataset—17,600 raw images; 25,000 after augmentation (7000 normal, 18,000 tumor images across 6 types)	5-fold validation
28.	Kumar et al. [42]	MRI (modality not specified)	253 brain MRI images (source not specified)	Not reported
29.	Dhaniya & Umamaheswari [43]	MRI (modality not specified)	UCI Machine Learning Lab (quantity not stated)	Not reported
30.	Montaha et al. [3]	Multimodal MRI (T1, T1c, T2, FLAIR), 3D volumes	BraTS 2018 (282 cases: 208 HGG, 74 LGG); BraTS 2019 (331 cases: 257 HGG, 74 LGG); BraTS 2020 (365 cases: 291 HGG, 74 LGG)	BraTS 2018 + 2019 combined for training/validation (361 subjects: 210 HGG, 151 LGG); BraTS 2020 used as external test set
31.	Muthaiyan & Malleswaran [44]	DICOM MRI (converted to BMP)	REMBRANDT—200 images (100 normal, 50 low-risk, 50 high-risk)	10-fold validation
32.	Yoo et al. [45]	Multimodal MRI (T1, T1c, T2, FLAIR), 2D axial slices (derived from 3D volumes)	BraTS 2020—369 3D volumes (~24 635 2D slices); BraTS 2023—886 2D slices (external test set)	BraTS 2020: 80% Training/10% Validation/10% Testing; BraTS 2023: External Validation
33.	Kuraparthi et al. [46]	2D MRI images (T1, T2, FLAIR—not specified)	Kaggle—253 images (2 classes: 98 no tumor, 155 tumor); BraTS 2015—332 images (2 classes: 156 LGG, 176 HGG)	70% Training/30% Testing for both datasets
34.	Park et al. [47]	Multimodal MRI (DWI/ADC, DSC/rCBV, DCE/Ktrans), 3T scanner	Clinical dataset, Severance Hospital, Yonsei University College of Medicine (South Korea)—129 patients with WHO grade II–III lower-grade gliomas	90 Training (2015–2019)/39 Testing (2012–2014); 10-fold cross-validation (100 replications) for feature selection
35.	Gates et al. [48]	Multimodal MRI (T1, T1c, T2, FLAIR, DWI, DSC, DCE, SWAN), 3T MRI	Prospective institutional dataset, MD Anderson Cancer Center (USA)—2323 patients (52 biopsy samples; 7 grade II, 9 grade III, 7 grade IV gliomas)	5-fold cross-validation; 80% Training/20% Testing

**Table 2:** Overview of machine learning methods used in brain tumor MRI analysis.

No.	Study	Method	Task	Approach and Preprocessing	Evaluation
1.	Mandle et al. [20]	K-means + SVM	Segmentation & Classification	Preprocessing with skull stripping, median filtering, and Otsu thresholding; segmentation via optimized K-means; feature extraction with DWT; feature selection using PCA; classification with K-SVM	Accuracy: 98.75%; Precision: 95.43%; Recall: 97.65%; Dice score for segmentation: 0.94; SSIM: 0.9901; MSE: 0.0012
2.	Wahlang et al. [21]	SVM	Classification (Normal vs. Abnormal)	Baseline comparison for classifying MRI brain images as normal/abnormal	Accuracy: 91%
		LeNet		LeNet-inspired CNN model; median filtering, image cropping, resizing to 194 × 194; included age/gender as additional input	Accuracy: 94%
		CNN-DNN		Cascade of CNN and deep dense layers; same preprocessing and demographic input as above	Accuracy: 95%
		ResNet50		Transfer learning using ResNet50; applied to resized MRI images.	Accuracy: 59%
3.	Sahaai et al. [12]	kNN	Multi-class Classification	Feature extraction using BRISK and shape/intensity descriptors; classification using kNN with Euclidean and other distance metrics	Accuracy: ~93–94%; Sensitivity: ~82–91%; slightly lower than SVM and RF
		SVM		BRISK-based feature extraction + image-based features; multi-class classificationusing SVM with BF kernel; optimizedvia grid search wih various kernels	Accuracy: 97.59%; Sensitivity: 93.24%; improved over kNN
		Random Forest		Classiication using RF enseble on BRISK + image-based features; 50 tres used in ensemble; outperformed SVM and kNN	Accuracy: 99.62%; Sensitivity: 99.16%; Specificity: 99.75%
4.	Yu et al. [22]	SVM	Tumor Type Classification (Glioma vs. Meningioma)	25 texture features extracted from MRI; feature selection via Gini index in RF; top 5 features used to train SVM classifier	AUC: 0.932; Sensitivity: 94.04%; Specificity: 92.3%; Error rate: 6.9%
		Random Forest		Feature selection using Gini impurity; classification model trained using RF on selected texture features	AUC: 0.856; Sensitivity: 82.8%; Specificity: 88.3%; Error rate: 14.1%
5.	Jo et al. [24]	Random Forest	Classification (treated vs. untreated meningiomas)	Semi-automatic segmentation; feature extraction using Pyradiomics; feature selection (Boruta + MRMR); lassification using RF with 10-fold cross-validation	AUC: 0.79; Accuracy: 73%; Sensitivity: 78.7%; Specificity: 67.4%
6.	Ni et al. [25]	LR, SVM, RF, NB, DT, GBT, XGB, LGBM	Classification (Ki-67 expression: low vs. high)	MRI preprocessing (registration, skull stripping); nnU-Net segmentation; radiomic feature extraction (PyRadiomics); feature selection via LASSO, correlation filter, and RF ranking; classification using multiple ML models with 10-fold cross-validation	Best: LR—AUC: 0.912; Accuracy: 0.881; SVM—AUC: 0.904; Accuracy: 0.884; RF—AUC: 0.882; Accuracy: 0.830
7.	Archana & Komarasamy [26]	U-Net	Segmentation (tumor localization)	Preprocessing steps included .MAT to .PNG conversion, cropping, and resizing of T1-weighted MRI images. U-Net was applied for tumor region segmentation across axial, coronal, and sagittal slices	Qualitative segmentation masks used for downstream classification (no Dice/IoU reported).
		Bagging-based KNN(BKNN)	Classification (glioma, meningioma, pituitary tumors)	Used segmented outputs from U-Net; BKNN classified tumor types using ensemble voting from multiple KNN models. Compared against KNN and AdaBoost + SVM baselines	BKNN accuracy: 97.7%; baseline KNN: 95.4%; AdaBoost + SVM: 96.3%; evaluated via confusion matrix and sensitivity/specificity per class
8.	Biswas & Islam [27]	Deep CNN + SVM	Classification (glioma, meningioma, pituitary tumors)	Image resizing, anisotropic diffusion filtering, adaptive histogram equalization; data augmentation; custom 5-layer CNN; SVM classification	Accuracy: 96.0%; Sensitivity: 95.71%; Specificity: 98.00%; Precision: 99.69%; F-measure: 96.92%
9.	Hashemzehi et al. [28]	CNN + NADE	Classification (glioma, meningioma, pituitary tumors)	Preprocessing of MRI images; feature extraction using CNN; distribution modeling and classification using NADE	Accuracy: 96.13%
10.	Papadomanolakis et al. [29]	DWT-CNN	Binary classification (tumor/no tumor)	MRI T2-SWI; wavelet transform DWT (3 levels); resolution 1320 × 15 × 20	Accuracy: 0.97; Sensitivity: 1.0; Specificity: 0.93; Precision: 0.95; FPR: 0.06; FNR: 0.0
		CNN		MRI T2-SWI; resolution 224 × 224; normalization; no feature processing	Accuracy: 0.97; Sensitivity: 0.94; Specificity: 1.0; Precision: 1.0; FPR: 0.0; FNR: 0.05
		CNN-TL (VGG16)		MRI T2-SWI; matched to the VGG16 input; TL from the ImageNet model	Accuracy: 0.87; Sensitivity: 0.91; Specificity: 0.84; Precision: 0.86; FPR: 0.4; FNR: 0.08
11.	Singh & Saxena [30]	2D CNN + Graph + Threshold	Classification and segmentation	MRI preprocessed (resized to 224 × 224, normalized); CNN trained for binary tumor/no-tumor classification; hybrid segmentation using graph-based (Felzenszwalb) and threshold methods	Accuracy: 98.89%; Bfscore: 1.0; Jaccard: 93.86%; Validation accuracy: 98.00%
12.	Pathak et al. [31]	CNN + Watershed segmentation	Classification and segmentation	MRI preprocessing with resizing and denoising; CNN for tumor/no-tumor classification; segmentation of tumors using marker-based watershed and morphological erosion	Accuracy: 98% (training), 100% (validation); Area calculation performed (16.56 mm^2^ tumor area)
13.	Badža & Barjaktarović [32]	CNN	Classification (glioma, meningioma, pituitary tumors)	Image normalization; resizing to 256 × 256; augmentation (rotation, flipping); custom 22-layer CNN in MATLAB	Accuracy: 96.56% (record-wise, augmented); 88.48% (subject-wise, augmented); Mean F1-score up to 97.47%
14.	Khan et al. [18]	CNN	Classification (binary)	Preprocessing via Canny edge detection and cropping; data augmentation (rotation, flip, brightness); CNN with 8 conv layers	Accuracy, Precision, Sensitivity, F1-score: 100%; AUC: 1.0
		VGG16			Accuracy: 90%; Precision: 93%; Sensitivity: 100%; F1-score: 97%; AUC: 0.96
		RenNet-50			Accuracy: 89%; Precision: 87%; Sensitivity: 93%; F1-score: 90%; AUC: 0.89
		Inception-v3			Accuracy: 75%; Precision: 77%; Sensitivity: 71%; F1-score: 74%; AUC: 0.75
15.	Kundari & Bhuvaneswari [33]	DCNN-SVM	Classification (glioma, meningioma, pituitary tumors)	Data augmentation (rotations, flipping, shifting, shear); feature extraction with LBP and ICA; SVM classifier	Accuracy: 98.96%; Sensitivity: 0.973;
16.	Yang et al. [34]	MUNet (U-Net + Mamba)	Segmentation	SD-SSM block for global-local fusion; SD-Conv for redundancy reduction; skip connections; mIoU, Dice, Boundary losses	Dice (BraTS2020): 0.835 (ET), 0.915 (WT), 0.823 (TC); Dice (BraTS2018): 0.835 (ET, TC), 0.901 (WT); Generalization validated on LGG
17.	Afzal et al. [6]	ResNet18 + Transfer Learning with CART-ANOVA	Multiclass classification (4 and 7 tumor types)	Median filtering, image resizing; transfer learning with ResNet18; CART-ANOVA hyperparameter tuning (LR, BS)	Accuracy: 99.65% (4-class), 98.05% (7-class); Validation accuracy on unseen dataset: 98.78% and 96.77%; F1-score: up to 99.69% (ResNet18)
18.	Ullah et al. [35]	CNN (from scratch), VGG-16, VGG-19, LeNet-5	Classification (binary)	Data augmentation (flipping, cropping, rotation); class balancing;training/test/validation split (70/20/10); hyperparameter tuning with Adam optimizer	VGG-16/VGG-19: Accuracy: 99.24%; Precision: 99%; Recall: 99%; Specificity: 99%; F1-score: 99%; CNN: 99.02%; LeNet-5: 98.80%
19.	Ullah et al. [36]	Inceptionresnetv2 (TL)	Classification	Pretrained models fine-tuned on Kaggle dataset; images resized and augmented via rotation/translation. No segmentation or feature extraction.	Accuracy: 98.91%; Precision: 98.28%; Recall: 99.75%; F1-score: 99.00%
		+ 8 other TL models (e.g., ResNet50, Xception, Mobilenetv2)		Same preprocessing pipeline for all models; performance compared to identify the best TL model.	Other models: Xception (98.37%); Mobilenetv2 (82.61%); ResNet101 (74.09%); ResNet50 (67.03%)
		Hybrid CNN + SVM		Deep features extracted from TL models, classified using SVM with linear kernel.	Accuracy: Mobilenetv2 + SVM = 98.5%, Densenet201 + SVM = 98.37%, ResNet101 + SVM = 98.01%
20.	Younis et al. [7]	CNN	Classification (binary)	Noise removal; bias correction; thresholding; resizing to 224 × 224	Accuracy: 96%; Recall: 89.5%; F1-score: 91.76%
		VGG16		TL; using feature maps from VGG16; resizing to 224 × 224	Accuracy: 98.5%; Recall: 94.5%, F1-score: 92.6%
		Ensemble (CNN + VGG)		Połączenie wyników CNN i VGG16; zmiana rozmiaru do 224 × 224	Accuracy: 98.14%; Recall: 91.4%; F1-score: 91.54%
21.	Zahoor et al. [37]	Ensemble CNN + SVM/MLP/AdaBoost (DBFS-EC); BRAIN-RENet + HOG + SVM (HFF-BTC)	Detection and Multi-class Classification	Phase 1: Ensemble of four TL-B CNNs (InceptionV3, ResNet-18, GoogleNet, DenseNet201) + SVM/MLP/AdaBoost; Phase 2: Fusion of BRAIN-RENet (dynamic features) and HOG (static) with SVM	Detection: Accuracy: 99.56%; F1-score: 0.9945; Classification: Accuracy: 99.2%; F1-score: 0.9909
22.	Preetha et al. [5]	EfficientNetB4 + Multi-Scale Attention U-Net	Segmentation	CLAHE, Gaussian blur, normalization, EfficientNetB4 encoder, multi-scale attention (1 × 1, 3 × 3, 5 × 5), residual attention blocks	Accuracy: 99.79%; Dice: 0.9339; IoU: 0.8795; Precision: 0.9657; Recall: 0.9103; Specificity: 0.9963
23.	Dixon et al. [38]	CNN + ViT + Radiomics + MLP	Multi-class classification (glioma, meningioma, pituitary, normal)	Preprocessing, augmentation (rotation, flip, contrast), feature extraction using GLCM, LBP, CNN, ViT; weighted feature fusion; MLP classifier	Accuracy: 99.19% (public); 99.38% (local); Sensitivity: 99.52%; Specificity: 99.53%
24.	Pacal et al. [2]	Swin Transformer + residual MLP for tumor classification	Classification (Multiclass)	Patch-based input; hybrid attention and residual MLP block; dataset augmentation via rotation, flipping, and scaling	Accuracy: 99.92%; F1-score: 99.89%; Precision: 99.93%; Recall: 99.84%
25.	Gajula &Rajesh [39]	CNN, SVM, RF, k-NN, NB, MLP	Multi-class classification (glioma, meningioma, pituitary, no tumor)	Adaptive filtering, global threshold segmentation, statistical and texture-based feature extraction	CNN: 98.6%; SVM: lower; AlexNet: 87.9%; VGG-16: 83.4%; RF/MLP/others: not explicitly detailed
26.	Shah et al. [40]	Transfer learning (EfficientNet-B0)	Classification	Preprocessing: grayscale conversion, blur, high-pass filtering; Data augmentation (Albumentations); Fine-tuning EfficientNet-B0 with custom FC layers	Accuracy: 98.87%; Precision: 0.989; Recall: 0.988; F1-score: 0.988; AUC: 0.988
		VGG16, InceptionV3, ResNet50		Transfer learning comparison with VGG16, ResNet50, IceptionV3	VGG16: 98.64%; InceptionV3: 97.5%; ResNet50: 95.8%
27.	Abd el Kader et al. [41]	Differential Deep-CNN	Classification	5-layer CNN with differential operators to expand feature maps; data augmentation to 25,000 MRI images	Accuracy: 99.25%; Sensitivity: 95.89%; Specificity: 93.75%; Precision: 97.22%; F1-score: 95.23%
28.	Kumar et al. [42]	GWDeepCNN-LSTM	Classification (tumor vs. normal)	Gaussian-weighted non-local mean filter, Hartigan’s clustering, Schutz index for similarity	Accuracy: 95%; Sensitivity: 98.21%; Specificity: 72.54%; MCC:0.8671; FPR = 5%
29.	Dhaniya & Umamaheswari [43]	CNN-LSTM	Classification (tumor vs. normal)	Wiener filtering; data augmentation (cropping, rotation, zooming, CLAHE, RRPS); segmentation using APSO; CNN for feature extraction; LSTM for classification	Accuracy: 92.03%; Sensitivity: 92.36%; Specificity: 91.42%; Precision: 92.93%; F-measure: 94.3
30.	Montaha et al. [3]	TD-CNN-LSTM	Classification (HGG vs. LGG)	Normalization (min-max) and resizing; 4 MRI sequences passed as input; TimeDistributed CNN for feature extraction; LSTM for sequence modeling; ablation study to optimize architecture	Accuracy: 98.90%; Precision: 98.95%; Recall: 98.78%; Specificity: 99.15%; F1-score: 98.83%; AUC: 99.04%
31.	Muthaiyan & Malleswaran [44]	Ensemble (SVM, NB, k-NN) + Bendlet	Classification (normal/abnormal, low/high risk)	Feature extraction using Bendlet transform with sub-band selection based on t-test; BCFs and histograms (HPBC and HNBC) were used for texture description; final classification was performed using an ensemble of SVM, Naive Bayes, and k-NN classifiers	Accuracy: 99.5%; Sensitivity: 99%; Specificity: 100%
32.	Yoo et al. [45]	Weakly Supervised CNN (AINet + ResNet-18)	Segmentation	Multimodal MRI (T1, T1c, T2, FLAIR); classifier + RISE seeds; deep superpixel generation and clustering trained on image-level labels; no manual segmentations	Dice: 0.745; HD95: 20.8 (BraTS 2023)
33.	Kuraparthi et al. [46]	AlexNet	Classification (binary)	Scaling images to 227 × 227 × 3; augmentation	Accuracy: 94.84% (Kaggle); 94.64% (BraTS)
		VGG16		Scaling images to 224 × 224 × 3; augmentation	Accuracy: 91.38% (Kaggle); 90.43% (BraTS)
		ResNet50		Scaling images to 224 × 224 × 3; augmentation	Accuracy: 98.23%; 97.87% (BraTS); AUC: 0.9978 (Kaggle); 0.9850 (BraTS)

## Data Availability

No new data were created or analyzed in this study. Data sharing is not applicable to this article.
